# Tissue Organoid Cultures Metabolize Dietary Carcinogens Proficiently and Are Effective Models for DNA Adduct Formation

**DOI:** 10.1021/acs.chemrestox.3c00255

**Published:** 2024-01-17

**Authors:** Angela L. Caipa Garcia, Jill E. Kucab, Halh Al-Serori, Rebekah S. S. Beck, Madjda Bellamri, Robert J. Turesky, John D. Groopman, Hayley E. Francies, Mathew J. Garnett, Meritxell Huch, Jarno Drost, Matthias Zilbauer, Volker M. Arlt, David H. Phillips

**Affiliations:** † Department of Analytical, Environmental and Forensic Sciences, School of Cancer & Pharmaceutical Sciences, King’s College London, London SE1 9NH, U.K.; ‡ Department of Medicinal Chemistry, Masonic Cancer Center, University of Minnesota, Minneapolis, Minnesota 55455, United States; § Department of Environmental Health and Engineering, Johns Hopkins Bloomberg School of Public Health, Baltimore, Maryland 21205, United States; ∥ Wellcome Sanger Institute, Cambridge CB10 1SA, U.K.; ⊥ Max Planck Institute of Molecular Cell Biology and Genetics, 01307 Dresden, Germany; # Princess Máxima Center for Pediatric Oncology, Oncode Institute, 3584 CS Utrecht, The Netherlands; ∇ Department of Paediatrics, University of Cambridge, Cambridge CB2 0QQ, U.K.

## Abstract

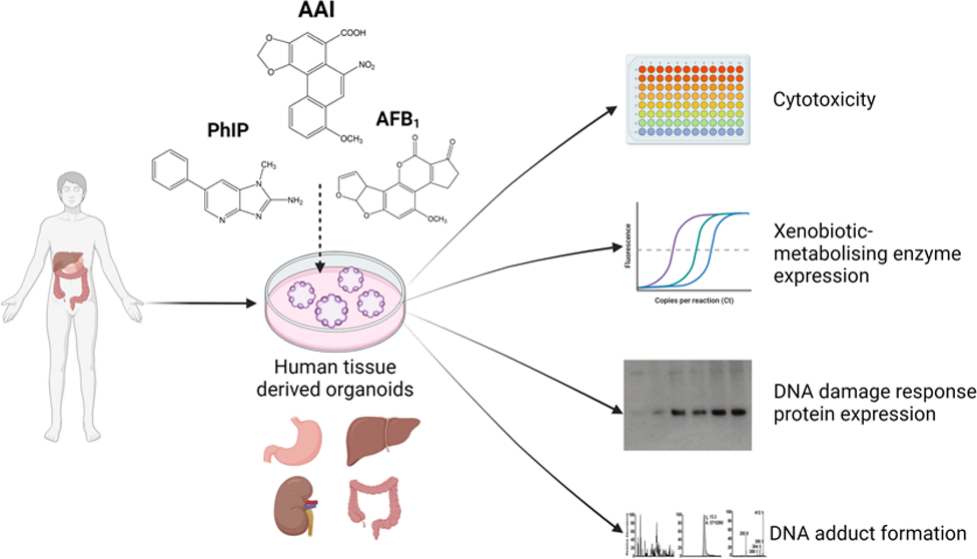

Human tissue three-dimensional (3D) organoid cultures have the potential to reproduce *in vitro* the physiological properties and cellular architecture of the organs from which they are derived. The ability of organoid cultures derived from human stomach, liver, kidney, and colon to metabolically activate three dietary carcinogens, aflatoxin B_1_ (AFB_1_), aristolochic acid I (AAI), and 2-amino-1-methyl-6-phenylimidazo[4,5-*b*]pyridine (PhIP), was investigated. In each case, the response of a target tissue (liver for AFB_1_; kidney for AAI; colon for PhIP) was compared with that of a nontarget tissue (gastric). After treatment cell viabilities were measured, DNA damage response (DDR) was determined by Western blotting for p-p53, p21, p-CHK2, and γ-H2AX, and DNA adduct formation was quantified by mass spectrometry. Induction of the key xenobiotic-metabolizing enzymes (XMEs) CYP1A1, CYP1A2, CYP3A4, and NQO1 was assessed by qRT-PCR. We found that organoids from different tissues can activate AAI, AFB_1_, and PhIP. In some cases, this metabolic potential varied between tissues and between different cultures of the same tissue. Similarly, variations in the levels of expression of XMEs were observed. At comparable levels of cytotoxicity, organoids derived from tissues that are considered targets for these carcinogens had higher levels of adduct formation than a nontarget tissue.

## Introduction

The potential of three-dimensional (3D) cell cultures in toxicological studies has been explored in large drug screens and by studying the biological and physiological effects of different xenobiotics [reviewed in ref (1)]. Organoids are 3D cultures derived from stem cells, embryonic, or adult tissue-resident cells that self-assemble into structures that consist of organ-specific cell types. They thus offer several potential advantages over other systems as they are derived from normal cells and can be used to reveal tissue-specific effects. They have been shown to partially recreate *in vitro* the cell organization and functions of the organ they derive from, including their expression of some xenobiotic-metabolizing enzymes (XMEs), making them potentially useful for the assessment of environmental carcinogens.^2,3^

There have, as yet, been few studies investigating the effects of environmental toxicants in organoids, and more research is needed in order to validate organoid models for their use in toxicology. We reported recently on the ability of human tissue organoids to metabolically activate the environmental carcinogen benzo[*a*]pyrene.^4^ Here, organoids from different human tissues were treated with three well-characterized dietary and environmental carcinogens, aflatoxin B_1_ (AFB_1_), aristolochic acid I (AAI), and 2-amino-1-methyl-6-phenylimidazo[4,5-*b*]pyridine (PhIP), in order to investigate the tissue-specific genotoxicity of these agents.

**Figure 1:**
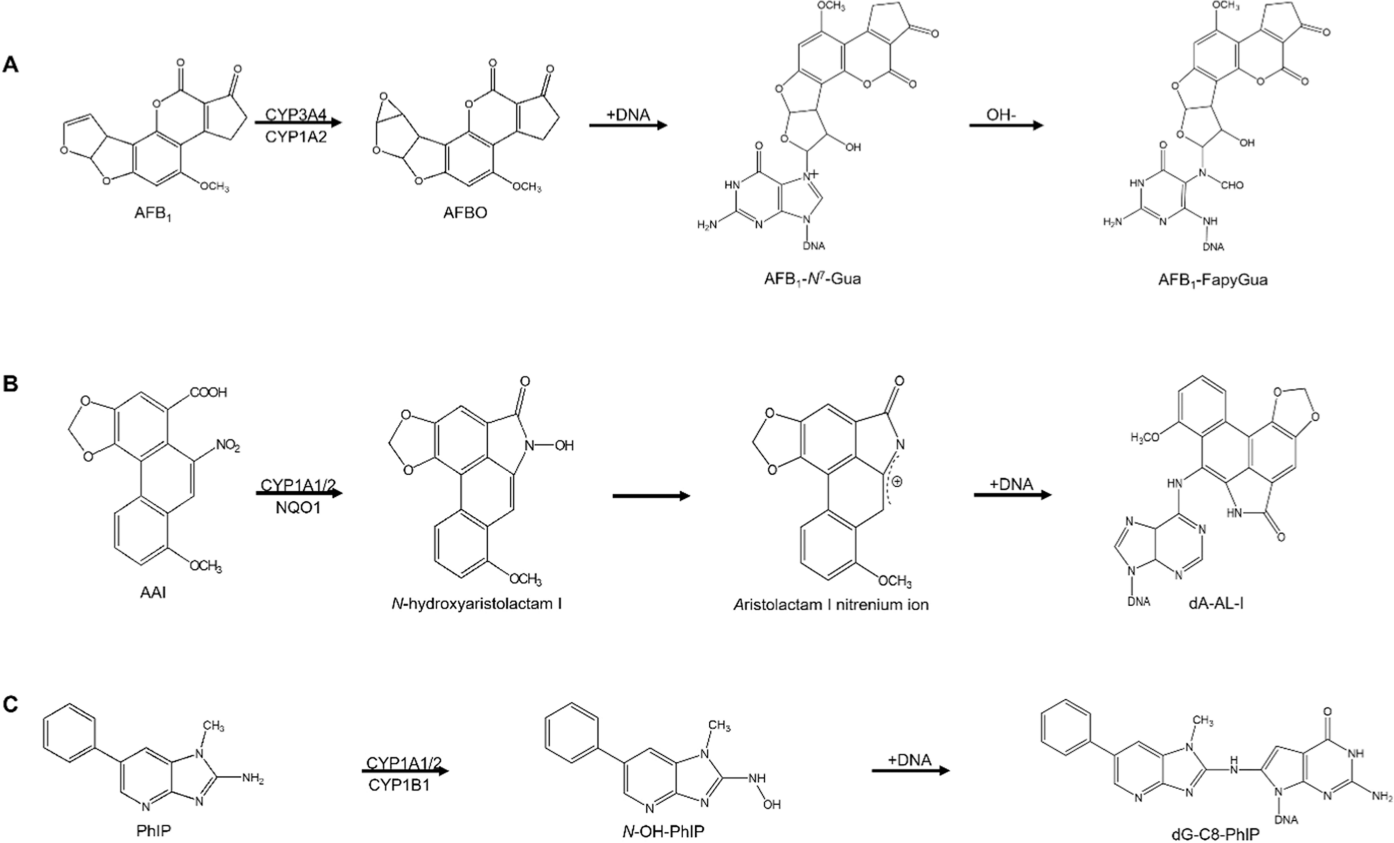
Pathways to the activation of the carcinogens in this study. (A) Aflatoxin B_1_ (AFB_1_); (B) aristolochic acid I (AAI); (C) 2-amino-1-methyl-6-phenylimidazo[4,5-*b*]pyridine (PhIP).

AFB_1_ is a mycotoxin mainly produced by the *Aspergillus flavus* and *Aspergillus parasiticus* fungi, which infect growing and poorly stored food crops, such as maize and nuts.^5^ AFB_1_ is a potent liver carcinogen that has been classified as a human carcinogen (Group 1) by the International Agency for Research on Cancer (IARC).^6,7^ AFB_1_ contamination is prevalent worldwide but highest in regions with high temperature and humidity, such as Southeast Asia and Sub-Saharan Africa. However, it is expected that due to climate change, additional regions will start to see significant contamination of crops with AFB_1_.^8,9^ The metabolic activation of AFB_1_ occurs in mammalian liver by the action of cytochrome P450 (CYP) enzymes, particularly CYP3A4 and CYP1A2, to produce the highly reactive metabolite AFB_1_-8,9-epoxide, which can bind to DNA to form the DNA adduct, 8,9-dihydro-8-(*N*^7^-guanyl)-9-hydroxy-AFB_1_ (AFB_1_-*N*^7^-Gua), which in turn can be converted to the persistent and highly mutagenic ring-opened adduct 8,9-dihydro-8-(2,6-diamino-4-oxo-3,4-dihydropyrimid-5-yl-formamido)-9-hydroxy-AFB_1_ (AFB_1_–FapyGua) (Figure 1A).^10−12^

AAI is the main component of the plant extract aristolochic acid (AA) from *Aristolochia* species, which are used in traditional herbal medicine for different purposes, including as an anti-inflammatory, a diuretic, an antiseptic, and to treat oedemas.^13−15^ AAI has been directly linked to chronic renal diseases, such as aristolochic acid nephropathy (AAN) and Balkan endemic nephropathy (BEN), which are associated with cancers of the upper urinary tract.^16,17^ The toxicity and carcinogenicity of AAI in humans has led to the banning of products containing it in most countries and to IARC classifying AA as a Group 1 human carcinogen.^13,14,18^ AAI undergoes metabolic activation to *N*-hydroxyaristolactam I that can react with DNA and form DNA adducts, mainly 7-(deoxyadenosin-*N*^6^-yl)-aristolactam I (dA-AL-I) (Figure 1B).^19^ This activation occurs principally by the action of NADPH:quinone oxidoreductase (NQO1) and CYP1A2, although CYP1A1 also plays a role in the bioactivation and detoxication of AAI.^20−24^

PhIP is a heterocyclic aromatic amine (HAA) that has been classified as a possible human carcinogen (Group 2B) by IARC.^25^ In experimental animals, PhIP induces tumors in multiple sites, including the colon,^26,27^ although there is no direct evidence that PhIP is a human carcinogen. The formation of PhIP and other aminoimidazoarene HAAs occurs through the Maillard reaction, which is a heat-catalyzed reaction of free amino acids, sugars, and creatine, a substance present in muscle cells.^28,29^ PhIP is the most abundant HAA formed in grilled beef, pork, fish, and poultry, and it is also a component of tobacco smoke.^30^ PhIP requires the action of XMEs to exert its genotoxicity. First, its exocyclic amine group is oxidized to produce *N*-hydroxy-2-amino-1-methyl-6-phenylimidazo[4,5-*b*]pyridine (*N*–OH-PhIP); this oxidation is catalyzed mainly by CYP1A2 in the liver, although CYP1A1 and CYP1B1 may contribute in other tissues.^31−33^ Although *N*–OH-PhIP can react with DNA, phase II activation involving *N*-acetyltransferases (NATs) and sulfotransferases (SULTs)—NAT2 and SULT1A1 are the most active^34^—further generates highly reactive esters, also leading to the formation of DNA adducts, mainly *N*-(deoxyguanosine-8-yl)-PhIP (dG-C8-PhIP) (Figure 1C).^32,33,35^

Here, organoids derived from four human tissues, stomach, liver, kidney, and colon were treated with AFB_1_, AAI, or PhIP, and cellular responses such as cytotoxicity, DNA damage response (DDR), and expression of XMEs were investigated, as well as the formation of DNA adducts. In each case, we compared a target tissue (liver for AFB_1_; kidney for AAI; colon for PhIP) with a nontarget tissue (gastric). The study contributes to efforts to introduce human organoids as testing systems in the field of environmental and genetic toxicology, as well as expand understanding of their metabolic competence.

## Materials and Methods

### Human Organoid Cultures

Gastric tissue from two donors (D88 and D95) was from the Wellcome Trust Sanger Institute, Hinxton, U.K., in accordance with the London Camden and King’s Cross Research Ethics Committee (REC#16/L0/1110). Liver tissue (one donor, D4) was from Addenbrooke’s Hospital, Cambridge, U.K., in accordance with the NRES Committee East of England—Cambridge Central (REC#16/EE/0227). Kidney tissue (two donors, D21 and D50) was from the Princess Maxima Centre for Pediatric Oncology, Utrecht, The Netherlands (#PMC2018–006JDR), in accordance with the Medical Ethical Committee of the Erasmus Medical Center (Rotterdam, The Netherlands; REC#MEC-2016–739). Colon tissue (two donors, D311 and D351) was from the Department of Paediatrics, University of Cambridge, Cambridge, U.K., in accordance with the East of England Cambridge South Research Ethics Committee (REC#17/EE/0265). Further details can be found in Supporting Table S1.

### Organoid Culture

Organoids were grown in 24-well plates, embedded in BME2 gel (Cultrex, #3533–010–02) or Matrigel (Corning, #356231), and overlaid with organoid type-specific growth medium (Supporting Table S2) as described.^36−40^ Growth medium was changed every 2–3 days. Organoids were passaged every 7–10 days, depending on density, by mechanical shearing or enzymatic digestion with TrypLE (Gibco, #12605028); after passaging organoid media was supplemented with 10 μM of the Rho-associated protein kinase (ROCK) inhibitor Y27632 (Stem Cell Technologies, #72308). Liver organoid differentiation into hepatocytes was carried out as reported.^36^

### Carcinogen Treatment

Stock solutions of AAI (Sigma, purity ≥97%) in sterile water at 20 mM, AFB_1_ (Sigma/Enzo, purity ≥98%) in dimethyl sulfoxide (DMSO) at 30 mM and PhIP (synthesized at the Biochemical Institute for Environmental Carcinogens, Lurup, Germany, purity ≥98%) in DMSO at 25 mM were stored in aliquots at −20 °C until use. Organoids were seeded in 96-well or 24-well plates 48 to 72 h before treatment. Stock solutions were diluted in organoid medium to the desired final concentrations, and the organoids were treated for 48 h. Solvent control cultures were treated with vehicle (water or DMSO) at the same final percentage as that used in the carcinogen treatments.

### Cell Viability Assessment

Stock organoid cultures at 60–80% density were disaggregated with TrypLE for 2–3 min at 37 °C and broken into small clumps (<25 cells per clump, approximately) by pipetting up and down. Disaggregated organoids were resuspended in BME2 at a dilution of 1:4–1:6 and seeded on 96-well plates (10 μL/well). Organoids were treated 2–3 days later. Cell viability after carcinogen treatment was measured using the CellTiter-Glo 3D Cell Viability Assay according to the manufacturer’s instructions (Promega, G9683). The reagent containing lysis chemicals, luciferin, and Ultra-Glo luciferase was added 48 h after treatment in a 1:2 ratio with the media. After 30 min incubation at room temperature, 50 μL was transferred to a white assay plate and luminescence was measured using a GloMax Explorer microplate reader (Promega). Each treatment was performed at least in triplicate (independent experiments).

### RT-qPCR

When organoids reached a density of 70–80% they were treated for 48 h with the carcinogens at concentrations that resulted in 50 and 80% viability. Organoids were harvested, and the pellets were lysed in 350 μL of RLT buffer (RNeasy Mini Kit; Qiagen, #74104) with 10 μL/mL β-mercaptoethanol. Lysates were homogenized by centrifuging through QIAshredder columns (Qiagen, #79654) for 2 min at 9600*g* and stored at −80 °C until RNA isolation. Total RNA was isolated using the RNeasy Mini Kit (Qiagen, no. 74104) according to the manufacturer’s instructions. RNA was measured using a NanoDrop 2000 Spectrophotometer. For cDNA synthesis, the High-Capacity RNA-to-cDNA Kit (Applied Biosystems, #4387406) or High-Capacity cDNA Reverse Transcription Kit (Applied Biosystems, #4368814) was used according to the manufacturer’s instructions. qPCR reactions were performed using TaqMan Gene Expression Master Mix (Applied Biosystems, #4369016) and Roche Universal Probe Library intron-spanning assays for the following NCBI sequences: NM_000499.3 (*CYP1A1*), NM_000903.2 (*NQO1*), NM_000761.5 (*CYP1A2*) and NM_017460.6 (*CYP3A4*). Each reaction was run at least in triplicate using ABI Fast optical 96-well (Applied Biosystems, no. 4346907) or 384-well reaction plates on an ABI Prism 7500 or 7900HT Fast Real-Time PCR machine (Applied Biosystems). Relative gene expression was normalized to the housekeeping gene *GAPDH* (NM_002046.5) and analyzed by the comparative threshold cycle method (*C*_t_). Results are reported as the relative fold change in expression (2^–ΔΔCt^) between the treated and solvent control samples.

### Western Blotting

When organoids reached a density of 70–80% they were treated for 48 h with the carcinogens at concentrations that resulted in 30, 50, and 80% viability. Organoids were then harvested and incubated in TrypLE for 10 min at 37 °C to remove the membrane matrix. The organoid pellet was washed with cold PBS and lysed in 62.5 mM Tris (pH 6.8), 1 mM EDTA (pH 8.0), 2% sodium dodecyl sulfate, 10% glycerol, 1× Halt Protease and Phosphatase Inhibitor Cocktail (Thermo Fisher Scientific, #78442). Western blotting was carried out as described.^41^ The primary antibodies used were: anti-p21 (1:2000; BD Bioscience, #BD556431), antiphospho-H2AX (Ser139, 1:1000; Cell Signaling, #9718S), antiphospho-CHK2 (T68, 1:1000; Cell Signaling, #2197S), antiphospho-p53 (Ser15, 1:2000; Cell Signaling, #9284S) and anti-GAPDH (1:25 000; Chemicon, #MAB374).

A lysate of human induced pluripotent stem cells (hiPSC) treated with 3.125 μM cisplatin was used as a positive control as previously described.^42^

### DNA Adduct Analysis

Organoids were harvested after treatment for 48 h and DNA was isolated using standard phenol-chloroform extraction.

For AFB_1_, samples were analyzed using liquid chromatography–tandem mass spectrometry (LC-MS/MS) at the Johns Hopkins Bloomberg School of Public Health.^43−45^ The internal standard AFB_1_–FapyGua-^15^N_5_ was added at 0.6 pmol to 3–30 μg of dried DNA sample. The samples were then dissolved in 100 μL of 0.1 M HCl and heated at 95 °C for 1 h. After cooling the samples, they were freeze-dried. The dried samples were dissolved in 100 μL of water before LC-MS/MS analysis.

A Thermo Scientific Finnigan TSQ Quantum Ultra AM triple quadrupole MS/MS system with an installed heated electrospray-ionization source was used for LC-MS/MS analysis. A Zorbax Extend C18 narrow-bore LC column (2.1 mm × 100 mm, 1.8 μm particle size) (Agilent Technologies, Wilmington, Delaware) with an attached Agilent Eclipse XDB-C8 guard column (2.1 mm × 12.5 mm, 5 μm particle size) was used to analyze the cis-AFB_1_–FapyGua and trans-AFB_1_–FapyGua adducts. The mobile phases used for analysis were: A: 98% water, 2% acetonitrile, and 0.1% formic acid, and B, acetonitrile with 0.1% formic acid. An initial gradient of 4% B/min starting from 100% A was used. After 10 min, mobile phase B was increased to 90% in 0.5 min and kept at this level for 5 min and then another 15 min at 8% to equilibrate the column. The flow rate was 0.3 mL/min, and the total analysis time was 25 min.

MS/MS was conducted using the following parameters: spray voltage at 3.5 kV; tube lens offsets at 89 V for Q1 and Q3; vaporizer temperature at 250 °C; capillary temperature at 340 °C; sheath gas (nitrogen) pressure at 50 arbitrary units; auxiliary gas (nitrogen) pressure at 30 arbitrary units; collision gas (argon) pressure at 6.67 × 10–5 Pa (5 mTorr). Selected reaction monitoring data were acquired in the positive ionization mode at a mass range of 100–1500 *m*/*z* with a scan width of 2.000 *m*/*z* and a scan time of 0.10 s. The data obtained were normalized to the amount of DNA used in the analysis. Adduct levels were calculated and expressed as adducts per 10^7^ bases.

For AAI and PhIP, samples were analyzed using ultraperformance liquid chromatography coupled with electrospray-ionization tandem mass spectrometry (UPLC-ESI/MS^3^) at the Masonic Cancer Center, University of Minnesota.^46^ Each DNA sample (5 μg) was resuspended in LC/MS water to a final volume of 94 μL and spiked with 2 μL of labeled internal standard (^15^N_3_-dA-AL-I and/or ^13^C_10_-dG-C8-PhIP) at a level of 5 adducts per 10^8^ nucleotides. To this were added 3.5 μL of Master mix I (2.5 μL of pH 7.1, 200 mM Bis-Tris and 1 μL of 1 M MgCl_2_) and 6 μL of Master mix II (5 μL of 1 mg/mL DNase I and 1 μL of 0.5 mg/mL nuclease P1), the samples then mixed gently by vortexing and incubated at 37 °C at 950 rpm for 4 h. After incubation, 3 μL of Master mix III (2 μL of 1 mg/mL alkaline phosphatase and 1 μL of 50 ng/mL phosphodiesterase I) was added and the samples incubated for 18 h at 37 °C at 950 rpm. After digestion, the DNA was dried under vacuum at room temperature, then resuspended in 28 μL of water:DMSO (1:1) and vortexed and sonicated for 5 min. This was followed by centrifugation for 10 min at 21,000*g*. 25 μL of each sample was transferred into an LC/MS vial.

DNA adducts, detected as modified nucleotides, were measured by UPLC-ESI/MS^3^ employing a Dionex Ultimate 3000 LC (Thermo Fisher, San Jose, California) equipped with a Thermo Acclaim PepMap trap cartridge RP C18 (0.3 mm × 5 mm, 5 μm particle size, 100 Å), a Michrom Magic C18 AQ column (0.1 mm × 150 mm, 3 μm particle size), and a Michrom Captive Spray source (Auburn, CA) interfaced with a linear ion trap mass spectrometer Velos Pro (Thermo Scientific, San Jose, CA). The LC mobile phases were (A) 0.01% formic acid and (B) 95% acetonitrile containing 0.01% formic acid. The DNA digest was injected onto the trapping column and washed with mobile phase A for 6 min at a flow rate of 12 μL/min. Then, the adducts were backflushed onto the Magic C18 AQ column at a flow rate of 1 μL/min. A linear gradient of 1–99% B over 10 min was applied for the separation, followed by 3 min of washing at 99% B. Analyses were conducted in the positive ionization mode. Optimized instrument tuning parameters were as follows: capillary temperature, 270 °C; source spray voltage, 1.8 kV; S-Lens RF level, 70%; injection time, 10 ms; activation Q, 0.35; and a normalized collision energy, 25 and 40 for MS^2^ and MS^3^, respectively. The ions monitored at the MS^3^ scan stage were as follows: dA-AL-I (543.3 *m*/*z* > 427.2 > 292.1, 293.1, 412.1), ^15^N_5_-dA-AL-I (548.3 *m*/*z* > 432.2 > 292.1, 293.1, 417.1), dG-C8-PhIP (490.1 *m*/*z* > 374.1 > 250.1, 304.1, 329.1, 357.1) and ^13^C_10_-dG-C8-PhIP (500.1 *m*/*z* > 379.1 > 251.1, 307.1, 333.1, 362.1). Adduct levels were calculated and expressed as adducts per 10^7^ nucleotides.

### Statistical Analysis

Results are shown as the mean ± SD. Sample size is indicated in each section. *GraphPad Prism* versions 8.4.3 and 9 (GraphPad Software Inc., La Jolla, CA) were used for statistical analyses. Relative mRNA expression data were log2 transformed with a one-sample *t*-test with Bonferroni correction against the control mean of 0 (*p < 0.05; **p < 0.01, difference from control).

## Results

### Cell Viability of Human Tissue Organoids

Cell viability after 48 h treatment with AFB_1_ (0–150 μM), AAI (0–200 μM), or PhIP (0–250 μM) was assessed using the CellTiter-Glo assay. Treatment with all carcinogens led to different levels of cytotoxicity between organoid types.

**Figure 2:**
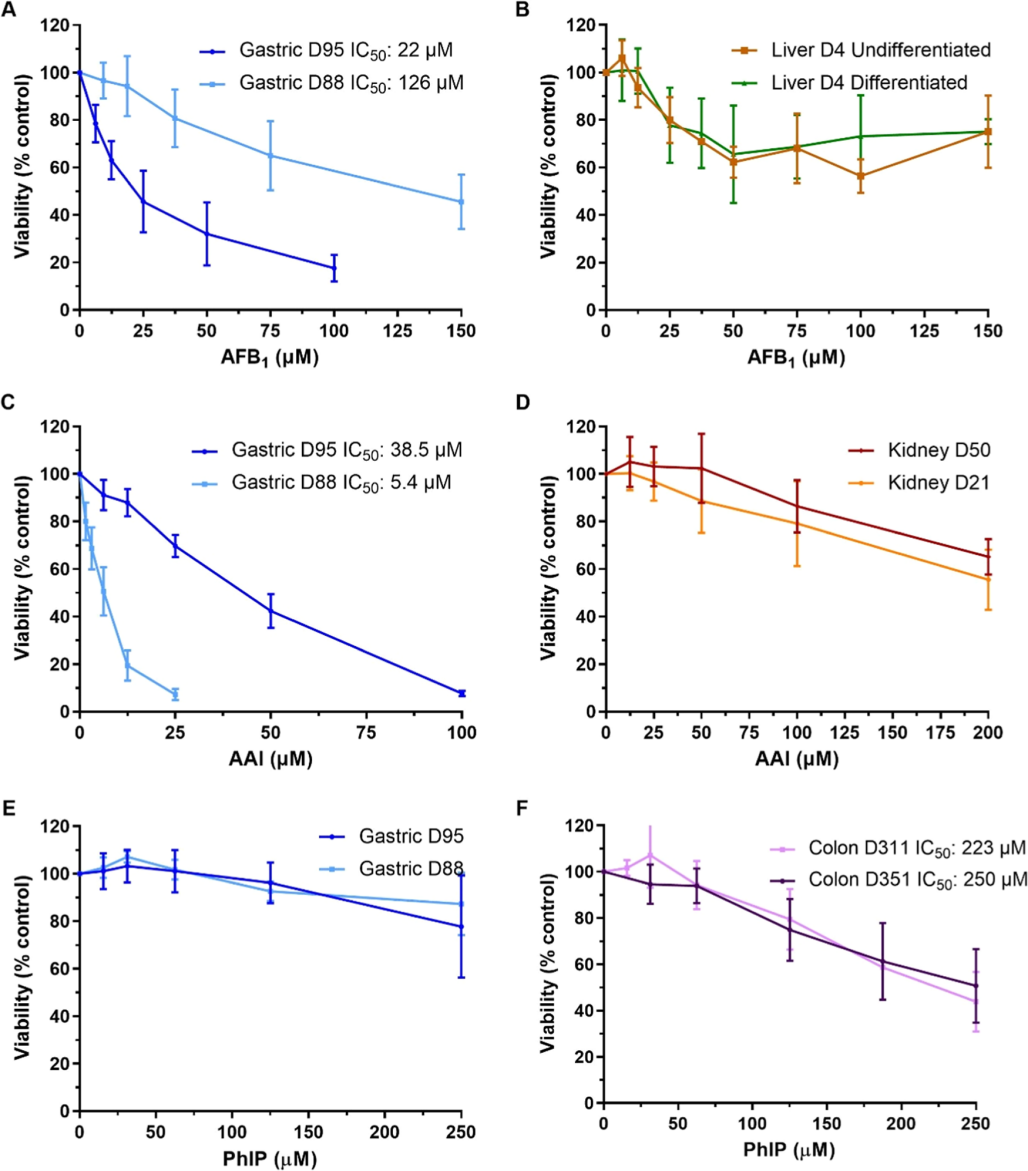
Cell viability in human tissue organoids treated with AFB_1_, AAI, and PhIP. Organoids from normal human stomach (A, C, and E; D95 and D88), liver (B; D4 undifferentiated and differentiated), kidney (D; D50 and D21), and colon (F; D351 and D311) tissues were treated with various concentrations of AFB_1_ (A, B), AAI (C, D), and PhIP (E, F) for 48 h. Vehicle controls DMSO (A, B, E, and F) or water (C, D) were included. Cell viability (% control) was measured using the CellTiter-Glo assay. Results are shown as mean ± SD (*n* ≥ 3).

The highest levels of cytotoxicity after AFB_1_ treatment were seen in gastric culture D95 with an IC_50_ of 22 μM, while gastric culture D88 had an IC_50_ of 126 μM. Both undifferentiated (*i.e.*, ductal) and differentiated (*i.e.*, hepatocyte) liver organoids showed a small decrease in cell viability with AFB_1_ but did not reach an IC_50_ (Figure 2A,B). AAI treatment led to a greater cytotoxic effect in gastric organoids than kidney organoids; gastric culture D88 was the most susceptible with an IC_50_ of 5.4 μM, followed by gastric D95 with an IC_50_ of 38.5 μM. Both kidney cultures had an estimated IC_50_ higher than 200 μM for AAI (Figure 2C,D). PhIP showed low levels of cytotoxicity; at the highest concentration tested (250 μM), only an ∼20% decrease in cell viability was measured in gastric organoids, while colon organoids had IC_50_ values close to this concentration (Figure 2E,F).

Based on these results, concentrations that induced 20–40% and 40–60% cell viability were chosen for subsequent experiments with each organoid culture. When there was no clear IC_50_, the highest concentration tested and a 2-fold lower concentration were selected.

### DDR Protein Induction

**Figure 3:**
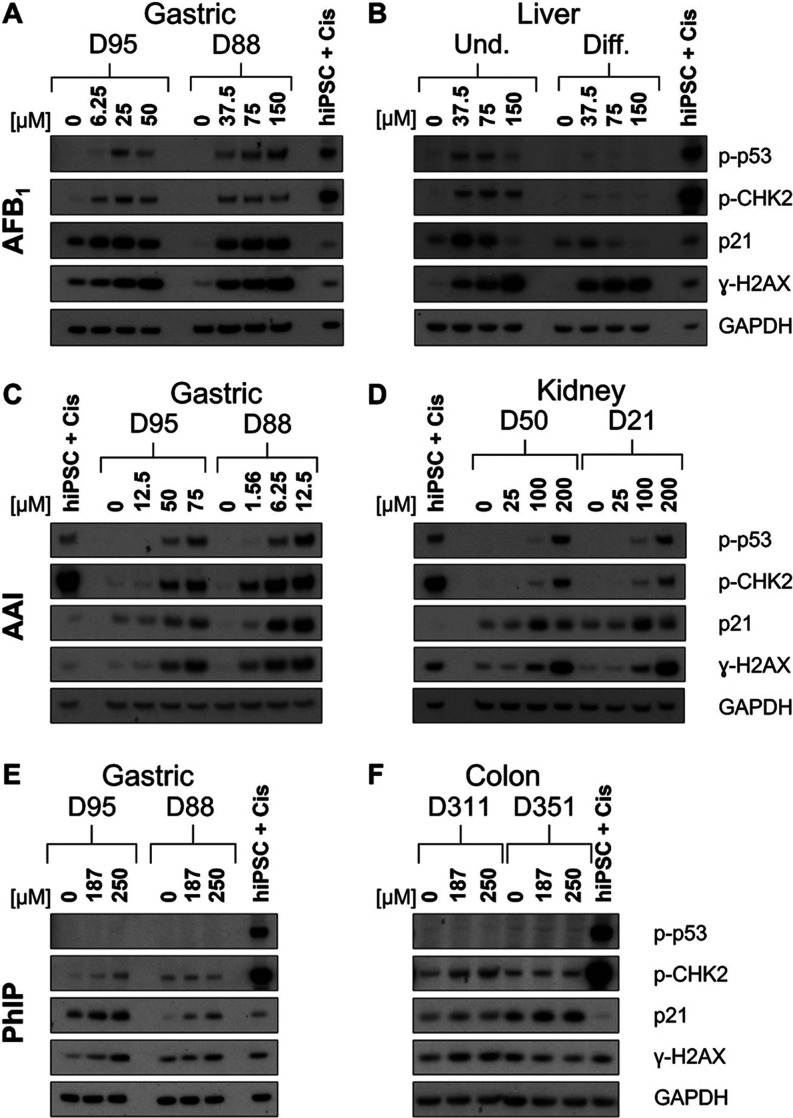
DDR in normal human tissue organoids treated with AFB_1_, AAI, and PhIP. Organoids from gastric (D95 and D88; A, C, and E), liver (D4 undifferentiated and differentiated; B), kidney (D50 and D21; D), and colon (D351 and D311; F) tissues were treated with the indicated concentrations of AFB_1_ (A, B), AAI (C, D), and PhIP (E, F) for 48 h, and lysates were analyzed by Western blotting. Various DDR proteins (p-p53, p-CHK2, p21, and γ-H2AX) were detected, and GAPDH was used as a loading control. iPSC + Cis (hiPSC treated with 3.125 μM cisplatin) was used as the positive control. Representative blots are shown (*n* = 2).

To further evaluate the effects of AFB_1_, AAI, and PhIP in organoids, induction of DDR proteins p-p53, p-CHK2, p21, and γ-H2AX was investigated by Western blotting. An additional concentration resulting in 60–80% viability was also examined. For AFB_1_, both gastric organoid cultures showed a dose-dependent increase in the level of γ-H2AX, while the other proteins seemed to have a higher induction at the lower concentrations compared to the highest treatment concentration. For D95, p-p53 and p-CHK2 had the highest induction at 25 μM, while p21 was most highly induced at 6.25 and 25 μM. For D88, the induction of p-CHK2 and p21 appeared constant across the concentrations tested, while p-p53 increased slightly at 150 μM (Figure 3A). Undifferentiated liver organoids had higher expression of these proteins than the differentiated organoids. Although γ-H2AX was induced in both differentiated and undifferentiated liver organoids, a stronger concentration-dependent increase was seen in the undifferentiated. As seen in other organoid types, p21 expression was the highest at 37.5 μM and then decreased to control levels at 150 μM. While undifferentiated organoids had induction of p-p53 at 37.5 and 75 μM, and p-CHK2 at all concentrations, p-p53 and p-CHK2 were weakly expressed in the differentiated liver (Figure 3B).

Treatment with AAI led to a dose-dependent response in gastric organoids. Both gastric organoid cultures displayed p-p53 induction at the two highest AAI concentrations. Expression of p-CHK2 and γ-H2AX increased at 50 and 75 μM in D95, while D88 had induction at all AAI concentrations. p21 was induced at the highest AAI concentrations in both gastric cultures, but the levels were higher in D88 (Figure 3C). Kidney organoids had clear induction of p-p53 and p-CHK2 only at 200 μM, although some expression can be seen at 100 μM. Expression of p21 was seen throughout; however, induction was seen only at 100 and 200 μM, with the highest expression occurring at 100 μM. γ-H2AX was also expressed in all conditions, and it was induced at 100 and 200 μM in a concentration-dependent manner (Figure 3D).

For PhIP, there was no induction of p-p53 in either the gastric or colon cultures. In gastric organoids, p-CHK2 expression was very low and induced only slightly by 250 μM PhIP in D95. p21 expression seemed to be concentration-dependent, with D95 having higher levels than D88 under all conditions. Similarly, γ-H2AX was induced in both donor gastric cultures, but the induction was stronger in D95 than in D88 (Figure 3E). In colon organoids, p-CHK2 and γ-H2AX were slightly induced only in D311, while in D351 p-CHK2 remained constant and γ-H2AX seemed to decrease. p21 was expressed and induced slightly in both donor cultures, but D351 had higher expression than D311 under all conditions (Figure 3F). Treatment of gastric organoids with the active metabolite of PhIP, *N*–OH-PhIP, showed a low induction of p-p53 in D95 at the highest concentration tested, and an induction of p-CHK2, γ-H2AX, and p21 in both D95 and D88 cultures (Supporting Figure S1).

### DNA Adduct Formation

**Figure 4:**
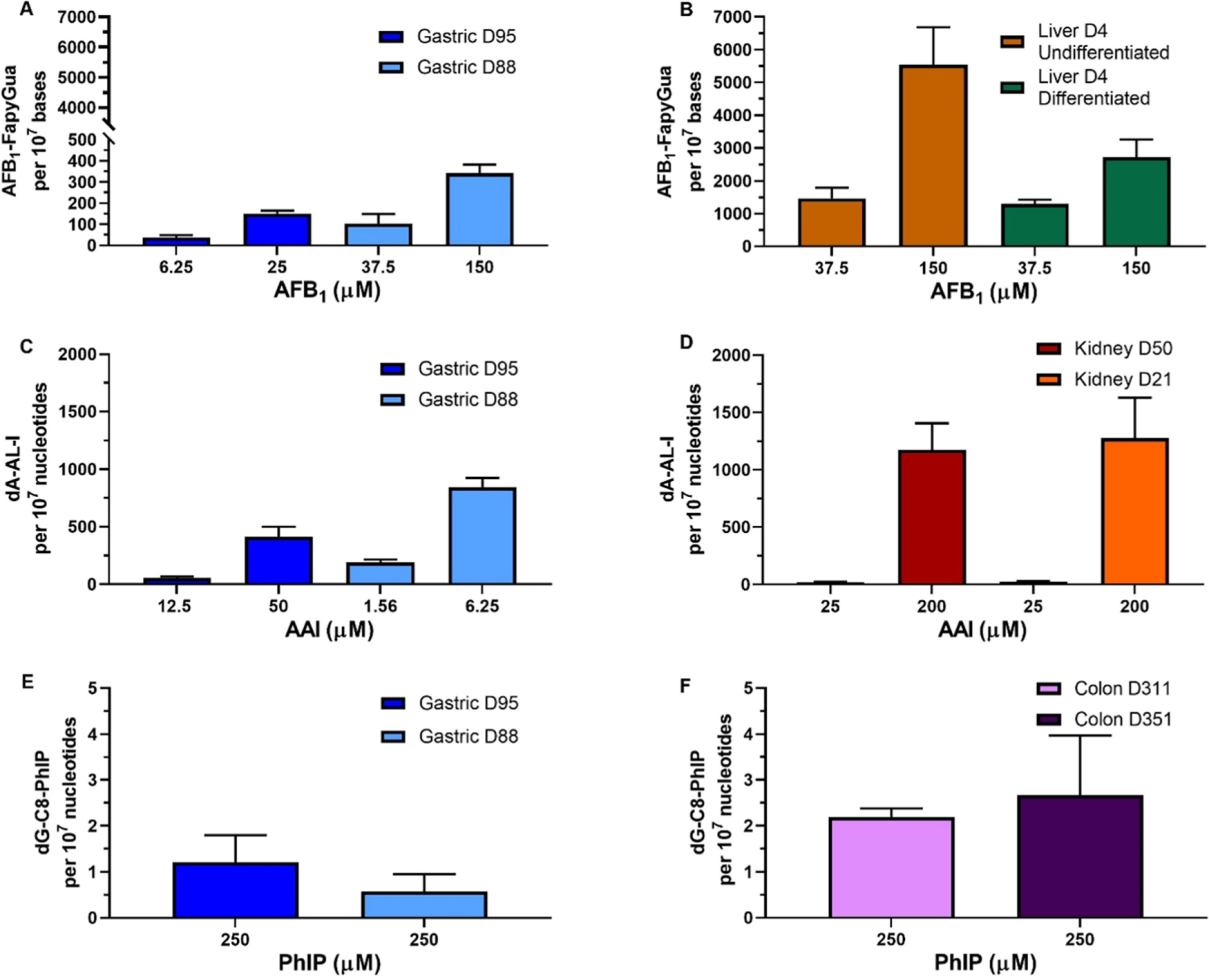
DNA adduct levels in human tissue organoids after treatment with AFB_1_, AAI, and PhIP. Gastric (D95 and D88; A, C, and E), liver undifferentiated and differentiated (D4; B), kidney (D50 and D21; D), and colon (D311 and D351; F) organoids were treated with the indicated concentrations of AFB_1_, AAI, and PhIP for 48 h. Vehicle controls (DMSO or water) were included (not shown). AFB_1_–FapyGua adduct was quantified using LC-MS/MS (A, B). dA-AL-I (C, D) and dG-C8-PhIP (E, F) adduct formation was quantified by using UPLC-ESI/MS^3^. Results are shown as mean ± SD (*n* ≥ 3).

To further evaluate the metabolic activation and genotoxicity of these dietary carcinogens, the formation of DNA adducts was investigated. The mean adduct levels for each compound in its treated organoids can be found in Supporting Table S3. After treatment with AFB_1_, levels of AFB_1_-*N*^7^-Gua adducts were low in all samples (data not shown); however, high levels of AFB_1_–FapyGua adducts, the ring-opened form derived from AFB_1_-*N*^7^-Gua, were observed. Overall, adduct formation in a concentration-dependent manner was seen in all organoid types, with the liver having a significantly higher level of adducts than the gastric and kidney organoids under all conditions tested (Figure 4A,B and Supporting Figure S2). Gastric organoids had the lowest levels of adducts of the three organoid types tested, with culture D95 having lower levels than D88. At the IC_50_ concentration, D95 had 151 adducts per 10^7^ nucleosides while D88 had 342 adducts per 10^7^ nucleosides. At the lower concentrations, D95 and D88 had 38 and 103 adducts per 10^7^ nucleosides, respectively (Figure 4A). Kidney organoids had much higher levels of adducts than the gastric and less differences between cultures. D50 had 1080 and 383 adducts per 10^7^ nucleosides at 150 and 37.5 μM, respectively, while D21 had 1177 and 426 adducts per 10^7^ nucleosides at the same concentrations. It is worth highlighting that the levels of cytotoxicity at 150 μM were slightly higher in the D50 culture (Supporting Figure S2). Lastly, the highest levels of AFB_1_–FapyGua adducts were detected in undifferentiated liver organoids, which had 5547 and 1467 adducts per 10^7^ nucleosides at 150 and 37.5 μM, respectively. Differentiated liver organoids also had high levels of adducts; however, at 150 μM, these were about half of those in the undifferentiated (2720 adducts per 10^7^ nucleosides). At 37.5 μM, differentiated liver organoids had 1298 adducts per 10^7^ nucleosides (Figure 4B).

Treatment with AAI led to the formation of dA-AL-I adducts in a concentration-dependent manner. Overall, gastric organoids had lower levels of adducts than kidney organoids at the highest concentration tested, with the kidney having 1.5- to 3-fold higher DNA adduct levels. It should be noted that the concentrations tested in kidneys were much higher than those in gastric organoids, as the concentrations used were determined by the cytotoxicity of the compound in each organoid. If considered relative to concentration, then adduct levels would be considered higher in gastric organoids. Higher levels of adducts were found in gastric D88 compared to gastric D95; at the lower concentrations tested, D88 had 193 adducts per 10^7^ nucleotides compared to 56 adducts per 10^7^ nucleotides in D95. At the IC_50_ concentrations, D95 and D88 had 412 and 843 adducts per 10^7^ nucleotides, respectively (Figure 4C). Although kidney D21 had slightly higher levels of adducts than that of D50, both kidney cultures had similar levels of adducts. At 25 μM they had around 20 adducts per 10^7^ nucleotides and at 200 μM around 1200 adducts per 10^7^ nucleotides (Figure 4D).

As the cytotoxicity and induction of DDR markers were low after PhIP treatment, DNA adduct formation was only assessed at 250 μM. Although dG-C8-PhIP adducts were detected at relatively low levels in all organoids, colon organoids had 2–4-fold higher levels than gastric organoids. Gastric D95 had higher levels than gastric D88 with 1.2 and 0.6 adducts per 10^7^ nucleotides, respectively (Figure 4E). Colon D351 showed slightly higher levels of adducts than D311, with 2.7 and 2.2 adducts per 10^7^ nucleotides, respectively (Figure 4F). In contrast, the active metabolite, *N*–OH-PhIP, led to higher levels of adduct formation in gastric organoids. Gastric D95 treatment with 2.5 and 3.75 μM resulted in 21.3 and 48.2 adducts per 1 × 10^7^ nucleotides, respectively. The levels of adducts in D88 were slightly higher, with 42.6 adducts per 10^7^ nucleotides at 17.5 μM and 56.8 adducts per 10^7^ nucleotides after treatment with 20 μM (Supporting Figure S1).

### Xenobiotic-Metabolizing Enzyme (XME) Expression

To further examine the activation of these compounds in the organoids and investigate the possible mechanisms of adduct formation, the mRNA expression levels of the main XMEs known to activate AFB_1_ and AAI were examined. Due to the low levels of adduct formation induced by PhIP treatment, XME analysis was not carried out for this compound.

**Figure 5:**
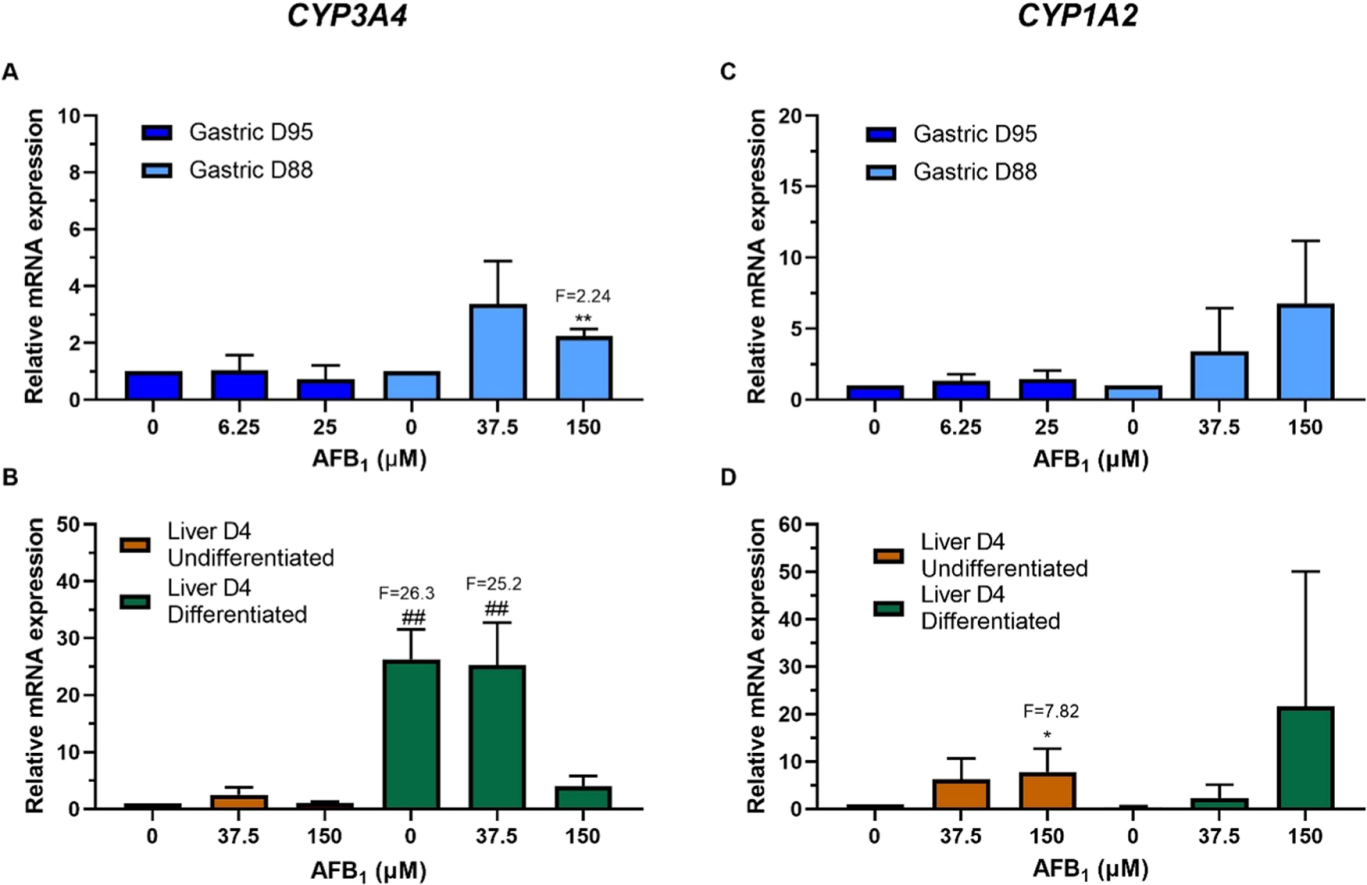
Relative gene expression of XMEs in human tissue organoids after AFB_1_ treatment. RT-qPCR and the 2^–ΔΔCT^ method were used to determine *CYP3A4* and *CYP1A2* expression in gastric (D95 and D88; A, C) and liver undifferentiated and differentiated (D4; B, D) organoids treated with the indicated AFB_1_ concentrations for 48 h. Values were normalized to mRNA expression of the housekeeping gene *GAPDH* and are relative to the vehicle control (0.5% DMSO); for liver organoids, the values are relative to the undifferentiated control. Results are shown as mean ± SD (*n* ≥ 3). Statistical analysis was performed by log 2 transforming the data and a one-sample *t*-test with Bonferroni correction against the control mean of 0: *p < 0.05; **p < 0.01 compared to untreated control; ^##^*p* < 0.01 compared to undifferentiated liver control.

AFB_1_ (*CYP3A4* and *CYP1A2*) and AAI (*CYP1A1*, *CYP1A2*, and *NQO1*) were examined by RT-qPCR. AFB_1_ treatment led to a significant induction of *CYP3A4* in gastric D88, but not D95, and in undifferentiated but not differentiated, liver. In gastric D88 the induction was significant at the higher concentration with a 2.2-fold increase (Figure 5A). In the case of the differentiated liver, the expression level of *CYP3A4* in the control was 26.3-fold higher than that of the undifferentiated control; after AFB_1_ treatment the expression seemed to decrease slightly, with a 25.2-fold increase at 37.5 μM compared to the undifferentiated control and no induction compared to the differentiated control (Figure 5B). Although *CYP1A2* expression increased in a concentration-dependent manner in all organoids after AFB_1_ treatment, induction was only significant in undifferentiated liver at 150 μM with a 7.8-fold change (Figure 5C,D).

**Figure 6:**
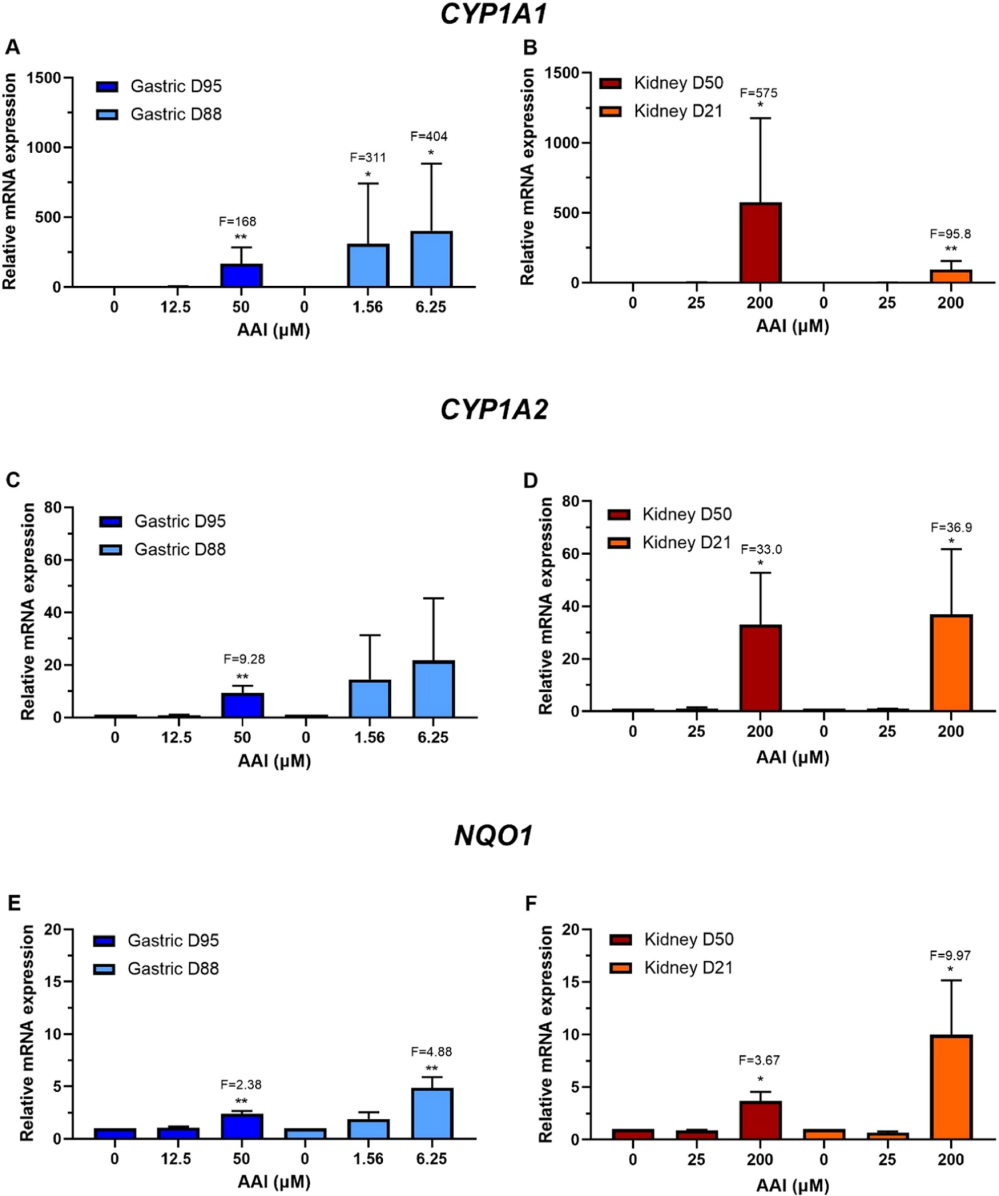
Relative gene expression of XMEs in human tissue organoids after AAI treatment. RT-qPCR and the 2^–ΔΔCT^ method were used to determine *CYP1A1*, *CYP1A2*, and *NQO1* expression in gastric (D95 and D88; A, C, and E) and kidney (D50 and D21; B, D, and F) organoids treated with the indicated AAI concentrations for 48 h. Values were normalized to mRNA expression of the housekeeping gene *GAPDH* and are relative to the vehicle control (0.1–1% water). Results are shown as mean ± SD (*n* ≥ 3). Statistical analysis was performed by log2 transforming the data and a one-sample *t*-test with Bonferroni correction against the control mean of 0 (*p < 0.05; **p < 0.01).

Treatment with AAI led to different induction levels of *CYP1A1*, *CYP1A2*, and *NQO1*. *CYP1A1* expression was significantly increased at the higher concentrations in all organoids, with gastric D95 and D88 having 168- and 404-fold inductions, respectively, and kidney D50 and D21 having 575- and 95.8-fold inductions, respectively (Figure 6A,B). *CYP1A2* induction was significant in gastric D95 at the higher concentration with 9.3-fold, and in both kidney cultures, which had similar levels at the higher concentration with 33- and 36.9-fold for D50 and D21, respectively (Figure 6C,D). *NQO1* expression was significantly induced at the higher concentrations used in all organoid cultures. Gastric D95 had slightly lower levels of induction than D88 (2.4-fold *versus* 4.9-fold). In the case of the kidney, D21 had higher expression levels (10-fold) than D50 (3.7-fold) (Figure 6E,F).

## Discussion

It is well known that many environmental carcinogens induce DNA damage, including DNA adduct formation; however, there are many other changes at the molecular level that have not been fully characterized for many of them. Studies in different *in vivo* and *in vitro* models have shed light on many of these processes. However, limitations such as species- and tissue-specific responses and the limited extent to which two-dimensional (2D) cell cultures reproduce physiological conditions *in vivo* have shown the need for better experimental models.^47,48^ This study addressed some of these limitations by investigating the metabolic activation of three dietary carcinogens and some key cellular responses to them in normal human tissue organoids.

The organoids used here were derived from normal tissue and consist of organ-specific epithelial cells and stem cells. Gastric organoids are mainly composed of gland mucus and chief cells.^38^ Undifferentiated liver organoids consist mainly of bile duct cells, and these differentiate into hepatocytes that carry out most of the metabolism of xenobiotics in this organ.^3,36^ Kidney organoids, also called tubuloids, consist of tubular epithelial cells that express proximal and distal tubule markers, as well as collecting duct markers and some markers for loop of Henle.^37^ Lastly, colon organoids are composed of intestinal epithelium purified from sigmoid colon fragments.^39^

Different cytotoxicity responses between organoid types and donor cultures were observed. Gastric organoid culture D95 was most susceptible to AFB_1_ toxicity with an IC_50_ almost 6-fold lower than that of the gastric D88 culture, while liver and kidney organoids were much less susceptible. Other *in vitro* studies of AFB_1_ with animal and human cells have shown reductions in cell viability ranging from small to large.^49−52^ However, many factors differed between these studies, including concentrations, exposure times, and methods used to measure cell viability, making comparisons complex.

Gastric organoids were also more susceptible than kidney organoids to AAI treatment, although the D95 gastric culture was much less sensitive than D88 cultures, having an IC_50_ for AAI around 6-fold higher. Chang et al.^53^ used a liver-kidney organ-on-chip to compare nephrotoxic responses with and without prior hepatic metabolism, showing that AAI-induced cell death in the kidney was significantly increased by prior hepatic metabolism. This could explain the low cytotoxic response seen in kidney organoids.

Colon organoids were more sensitive to PhIP than gastric organoids, although the compound was relatively noncytotoxic in both, compared with AFB1 and AAI. PhIP treatment led to a decrease in colon organoid cell viability of approximately 50% at the highest concentration tested (250 μM), while gastric organoids had only a small decrease in cell viability at the same high concentration. These results correlate with previous studies of PhIP in human and mouse cell lines from different tissue origins (colon, liver, breast, and lung) showing low cytotoxicity in both 2D and 3D cultures (HepG2 spheroids) in the absence of S9.^41,49,54−56^ In several studies, coculture with metabolically active cells or with the addition of S9 mix was required to see an effect.^41,42,55^

Bulky DNA adducts, such as those formed by all three of the compounds studied here, are repaired by the nucleotide excision repair (NER) pathway.^57,58^ It has been shown that NER can lead to the formation of single- and double-strand breaks, which trigger the phosphorylation of H2AX and the activation of the ATM and ATR pathways.^59^ Therefore, here the expression of proteins involved in these processes such as p-p53 and p21 was examined; the latter proteins are also involved in the maintenance of the G1/S checkpoint, p-CHK2, and γ-H2AX. AFB_1_ treatment led to the induction of p-p53 and p-CHK2, which indicates the activation of the ATM pathway, a response previously seen following treatment of cells with AFB_1_.^60^ Gastric D88 had a concentration-dependent induction of p-p53 and a constant level of induction of p-CHK2 and p21; in the other organoid lines, the highest induction of p21, p-p53, and p-CHK2 was seen at the lowest concentrations. Lower expression of these markers at the highest concentration may be due to increased toxicity; however, the precise reason is unclear. Previous studies have reported induction of these DDR markers after AFB_1_ treatment, but a lack of CHK2 and p53 phosphorylation has also been seen,^42,50,61−63^ which correlates with the nonactivation of p53 and CHK2 at higher concentrations in differentiated liver organoids in the present study. In the present study, a concentration-dependent induction of γ-H2AX in all organoid lines treated with AFB_1_ was observed.

The DDR response to AAI treatment led to higher levels of induction in gastric D88 than in D95. Both kidney organoid cultures had similar levels of induction of all proteins. AAI treatment induced p-CHK2, particularly at the highest concentrations, as previously reported in human HK-2 kidney proximal tubular cells.^64^ Induction of γ-H2AX was also seen. Furthermore, gastric organoids showed a concentration-dependent induction of both p-p53 and p21, while kidney organoids showed concentration-dependent induction of p-p53, but induction of p21 only at 100 μM AAI. These findings agree with previous reports of induction of p-p53 and p21 by AAI in other experimental models.^65−69^

The low induction of DDR by PhIP matched the low cytotoxic responses and relatively low levels of dG-C8-PhIP adducts detected in the organoids. Small inductions of p21, p-CHK2, and γ-H2AX were seen in some gastric and colon organoid cultures; gastric D95 and colon D311 had stronger responses than gastric D88 and colon D351, respectively. Induction of p21 and γ-H2AX by PhIP has been observed previously in human and hamster cells.^55,70^ Although Yang et al.^71^ showed that p53 is activated after PhIP treatment in mice, no induction of p-p53 was seen here in organoids. This correlates with findings by Hölzl-Armstrong et al.,^41^ where murine FE1 lung epithelial cells treated with PhIP without the addition of S9 mix had no induction of DDR markers. Notably, we did measure the induction of all DDR markers in gastric D95 organoids after treatment with the PhIP reactive metabolite, *N*–OH-PhIP.

Importantly, evidence of DNA adduct formation demonstrates the metabolic activation of carcinogens in the organoids. Low levels of AFB_1_-*N*^7^-Gua were seen compared to AFB_1_–FapyGua adducts, which were detected in all cultures. It has been shown that the initial adduct AFB_1_-*N*^7^-Gua, half-life ∼7.5 h, is gradually converted into the persistent AFB_1_–FapyGua adduct, with approximately 20% of the original adduct converted after 24 h and 70% removed from the DNA,^72^ and approximately 10% of the peak level of AFB_1_-*N*^7^-Gua adducts remaining after 48 h.^45^ This was also seen in the livers of new-born mice injected with AFB_1_, where there was a significant decrease in AFB_1_-*N*^7^-Gua adducts between 6 and 48 h and higher levels of AFB_1_–FapyGua than AFB_1_-*N*^7^-Gua were detected at 48 h.^73^ Thus, after 48 h treatment of organoids, only a small amount of initially formed AFB_1_-*N*^7^-Gua remains. The highest levels of AFB_1_–FapyGua adducts were detected in liver organoids, intermediate in kidneys, and lowest in gastric. This finding is in line with a rat study in which the levels of AFB_1_-*N*^7^-Gua adducts were much higher in liver than in kidney and other tissues including colon.^74^ Similarly, AFB_1_–FapyGua adducts have been found in liver of human subjects at higher levels than in colon, rectum, pancreas, breast, and cervix.^75^

AFB_1_ activation in the organoids was also supported by the induction of XMEs involved in this process. As the hepatocytes carry out the main metabolic activities of the liver, it was expected that the undifferentiated liver organoids (primarily ductal cells) would have lower induction levels of the CYP enzymes than the differentiated liver organoids (containing hepatocytes) after AFB_1_ treatment. This was the case for both CYP enzymes examined; however, because the induction of *CYP1A2* in differentiated liver had high variability, the difference did not reach statistical significance. Although gastric culture D95 had the highest cytotoxic response to AFB_1_, it had the lowest levels of induction of both *CYP1A2* and *CYP3A4*, followed by gastric D88. Interestingly, *CYP3A4* induction was not concentration-dependent, with the highest induction seen at the lowest AFB_1_ concentration tested, while *CYP1A2* expression followed a concentration-dependent pattern. Several studies have looked at the involvement of these enzymes in AFB_1_ metabolism;^76,77^ however, investigations of the effects of AFB_1_ treatment on their expression in different tissues or cell types have been limited. Induction of these XMEs in HepG2 spheroids has been reported to be only small.^49^

The levels of dA-AL-I adducts correlated with the levels of expression of the DDR markers and XMEs, which were induced in a concentration-dependent manner. Interestingly, kidney organoids had higher adduct levels at the highest concentration tested, although they had a lower cytotoxic response, which indicates toxicity may not be caused solely by adduct levels. Gastric D88 had higher levels of AAI-DNA adducts and XMEs expression than D95, which correlated with the greater susceptibility of D88 to AAI cytotoxicity. Similarly, the more susceptible kidney culture, D21, had higher induction of *NQO1* and *CYP1A2* than D50 at 200 μM, although *CYP1A1* expression was much higher in D50 than in D21. Kidney organoids had similar levels of DNA adducts at both concentrations tested; however, D21 had slightly more adducts than D50. Although the induction of *NQO1*, the main AAI activating enzyme, was higher in D21, the levels of *CYP1A1* were higher in D50. The high expression of *CYP1A1* may account for the similar levels of adducts in both D21 and D50 to some extent as this enzyme is also involved in the detoxication of AAI.^78,79^ High levels of dA-AL-I adduct formation in kidney tissues of humans and animals have been reported in numerous studies.^78,80−82^ Adduct formation has also been seen in the glandular stomach and forestomach of rodents,^81,82^ but at levels lower than in the kidney and there was little evidence of AA-DNA adduct formation in human stomach,^83^ consistent with the higher adduct levels in kidney organoids than in gastric organoids seen here. Induction of protein and gene expression as well as activity of these XMEs after AAI treatment has been reported in various models.^65−68,82,84,85^

DNA adduct formation in PhIP-treated gastric organoids followed the same pattern seen in the cytotoxicity experiments, where organoids with greater cytotoxicity had a higher level of adducts. Gastric culture D95 had levels of dG-C8-PhIP higher than D88. Interestingly, although colon organoids had very similar levels of dG-C8-PhIP, colon culture D351 had higher adduct levels than did D311, despite D311 having slightly stronger cytotoxic and DDR responses. Colon organoids formed higher levels of adduct than gastric organoids, in line with studies in mice where PhIP-DNA adduct formation was higher in the colon than in the glandular stomach.^86,87^ Gastric organoids had a greater response to *N*–OH-PhIP than to PhIP and, as seen with PhIP, D95 was more susceptible to *N*–OH-PhIP than D88. This agrees with previous studies where cell viability was not affected after PhIP treatment, while *N*–OH-PhIP at the same or lower concentrations led to a decrease in viability.^41,88^

A recent study^4^ reported the ability of human organoids derived from normal gastric, pancreas, liver, colon, and kidney tissues to metabolize the environmental carcinogen benzo[*a*]pyrene (BaP), accompanied by activation of the DDR pathway (induction of p-p53, p-CHK2, p21, and γ-H2AX) and by DNA adduct formation. Organoids from the different tissues showed varied cytotoxic responses to BaP, with upregulation of XME genes. Additionally, high-throughput RT-qPCR revealed differences in gene expression among organoid types after BaP treatment. Taken together with the present study, these results demonstrate the potential usefulness of organoids for studying environmental carcinogenesis and genetic toxicology.

It should be noted that donors of different ages provided organoids for this study. It is possible that age of donor is a factor in the variations observed, although gender and culture conditions (serum, growth factors, and inhibitors) may also be influential factors. Further studies will be required to ascertain the extent of such influences, although it can at least be stated that the differences observed here are, for the most part, quantitative rather than qualitative.

In conclusion, organoids from multiple tissues have the metabolic capability to activate AAI, AFB_1_, and PhIP to different extents. Moreover, in some cases, this metabolic potential varied between different donor cultures of the same tissue. Similarly, high levels of variation in the expression of XMEs between organoid types and, in some cases, between donor cultures were observed. Organoids derived from tissues that are considered targets of tumor formation for these carcinogens had higher levels of adduct formation than a nontarget; albeit a single comparator in two cases and two nontargets in the other. Further studies will be required to ascertain whether this trend is consistent. Thus, far, the focus of this study has been on early events in carcinogenesis. Organoids should also be of use in the investigation of further molecular changes after treatment with carcinogens, such as gene expression and epigenetic changes. While issues in standardization of organoids may limit their use for routine or regulatory genetic toxicology, they may be of value in mechanistic studies to determine the relevance of rodent experiments to humans. An advantage of organoids is that they are essentially primary cells with the capacity of renewal, and they partially recreate *in vitro* the cell organization and functions of the organs from which they are derived. It can be envisaged that mechanistic insights and interspecies extrapolations could be obtained on a case-by-case basis by comparing, for example, the responses of rodent and human organoids derived from the organ in which tumors have been induced in an animal bioassay.

## Supporting Information Available

Organoids from different human tissues (Table S1); growth media recipes for organoids derived from different human tissues (Table S2); DNA adduct levels in normal human tissue organoids after treatment with AFB1, AAI, PhIP, and *N*–OH-PhIP (Table S3); organoids from normal human gastric tissues (D95 and D88) were treated with various concentrations of *N*–OH-PhIP for 48 h (Figure S1); and organoids from normal human kidney tissues (D50 and D21) treated with various concentrations of AFB1 for 48 h (Figure S2) (PDF)

## Abbreviations

3D: three-dimensional
XMEs: xenobiotic-metabolizing enzymes
AFB1: aflatoxin B1
AAI: aristolochic acid I
PhIP: 2-amino-1-methyl-6-phenylimidazo[4,5-*b*]pyridine
CYP: cytochrome P450
IARC: International Agency for Research on Cancer
AFB1-N7-Gua: 8,9-dihydro-8-(N7-guanyl)-9-hydroxy-AFB1
AFB1-FapyGua: 8,9-dihydro-8-(2,6-diamino-4-oxo-3,4-dihydropyrimid-5-yl-formamido)-9-hydroxy-AFB1
dA-AL-I: 7-(deoxyadenosin-N6-yl)-aristolactam I
NQO1: NADPH:quinone oxidoreductase
dG-C8-PhIP: *N*-(deoxyguanosine-8-yl)-PhIP
DMSO: dimethyl sulfoxide
