# Supplementary material for: Tissue Organoid Cultures Metabolize Dietary Carcinogens Proficiently and Are Effective Models for DNA Adduct Formation
Source: Chem Res Toxicol. 2024 Jan 17;37(2):234–47. doi: 10.1021/acs.chemrestox.3c00255 (PMC10880098; doi:10.1021/acs.chemrestox.3c00255)
Supplement: Supplementary file 1 — tx3c00255_si_001.pdf [file tx3c00255_si_001.pdf]

Tissue organoid cultures metabolise dietary carcinogens proficiently and are effective models for DNA adduct formation

Supplementary Information

Angela L. Caipa Garcia[1,*], Jill E. Kucab[1], Halh Al-Serori[1], Rebekah S.S. Beck[1], Madjda Bellamri[2], Robert J. Turesky[2], John D. Groopman[3], Hayley E. Francies[4], Mathew J. Garnett[4], Meritxell Huch[5], Jarno Drost[6], Matthias Zilbauer[7], Volker M. Arlt[1,8] and David H. Phillips[1, *]

[1] Department of Analytical, Environmental and Forensic Sciences, School of Cancer & Pharmaceutical Sciences, King's College London, London, SE1 9NH, UK

[2] Department of Medicinal Chemistry, Masonic Cancer Center, University of Minnesota, Minneapolis, MN 55455, USA

[3] Department of Environmental Health and Engineering, Johns Hopkins Bloomberg School of Public Health, Baltimore, MD 21205, USA

[4] Wellcome Sanger Institute, Cambridge CB10 1SA, UK

[5] Max Planck Institute of Molecular Cell Biology and Genetics, 01307 Dresden, Germany

[6] Princess Máxima Center for Pediatric Oncology, Oncode Institute, 3584 CS Utrecht, The Netherlands

[7] Department of Paediatrics, University of Cambridge, Cambridge, CB2 0QQ, UK

[8] Present address: Toxicology Department, GAB Consulting GmbH, 69126 Heidelberg, Germany

* To whom correspondence should be addressed. Tel: +44 (0)20 7848 4569; Email: david.phillips@kcl.ac.uk.

Correspondence may also be addressed to Angela Caipa Garcia, Tel: +44 7761 319185; Email: angela.caipa_garcia@kcl.ac.uk

S2 **Supplementary Table S1.** Information about organoids from different human tissues

S3 **Supplementary Table S2.** Growth media recipes for organoids derived from different human tissues

S5 **Supplementary Table S3.** DNA adduct levels in normal human tissue organoids after treatment with AFB_1, AAI, PhIP and *N*-OH-PhIP

S6 **Supplementary Fig. S1.**

S7 **Supplementary Fig. S2.**

Supplementary Table S1. Information about organoids from different human tissues

Tissue	Donor ID	Age	Gender	Specific biopsy information	Collaborating research team	Research Ethics Committee (REC)	REC #
Gastric	D88	76	Male	Normal gastric tissue from the upper stomach was removed from two patients (Donor 88 and Donor 95) that were undergoing biopsy for an oesophageal tumour. Organoid cultures were derived from the tissue at the Sanger Institute as previously described [89].	Hayley Francies/ Mathew Garnett (Wellcome Trust Sanger Institute)	London - Camden and Kings Cross Research Ethics Committee	16/L0/1110
	D95	69	Male				
Colon	SC311 (D311)	6	Male	Normal sigmoid colon tissue taken by endoscopy from healthy, control donors [40].	Matthias Zilbauer (Department of Paediatrics, University of Cambridge)	East of England Cambridge South Research Ethics Committee	17/EE/0265
	SC351 (D351)	11	Female				
Kidney	JD021 (D21)	3	Female	Normal tissue was taken following nephrectomy or biopsy [37].	Jarno Drost (Princess Maxima Centre for Pediatric Oncology, Utrecht, The Netherlands)	Medical ethical committee of the Erasmus Medical Center (Rotterdam, the Netherlands).	MEC-2016-739
	JD050 (D50)	4	Female				
Liver	D4	Unknown	Female	Biopsies of normal liver tissue were obtained from deceased patients during surgery for organ transplantation [3].	Meritxell Huch (Max Planck Institute of Molecular Cell Biology and Genetics)/ Kourosh Saeb-Parsy (Department of Surgery, Addenbrookes Hospital)	NRES Committee East of England - Cambridge Central	16/EE/0227

Supplementary Table S2. Growth media recipes for organoids derived from different human tissues

Medium component	Final concentration						Source
	Gastric	Liver	Kidney	Colon	Liver Starting	Liver Differentiation	
Advanced DMEM/F12	√	√	√	√	√	√	Life technologies, #12634-010
HEPES	10 mM	10 mM	10 mM	10 mM	10 mM	10 mM	Life Technologies, #15630-056
Glutamax	1X	1X	1X	1X	1X	1X	Life technologies, #35050-038
WNT3A CM	50%	-	-	50%	30%	-	ATCC, CRL-2647
R-Spondin-1 CM	10%	10%	10%	20%	10%	-	Cultrex, #3710-001-01
N-acetyl cysteine	1.25 mM	1.25 mM	1 mM	1.25 mM	1.25 mM	1.25 mM	Sigma, #A9165
Human Noggin	150 ng/mL	-	-	-	25 ng/mL	-	Peprotech, #120-10C
Mouse Noggin	-	-	-	100 ng/mL	-	-	Peprotech, #250-38-250ug
Recombinant Human EGF	50 ng/mL	50 ng/mL	50 ng/mL	-	50 ng/mL	50 ng/mL	Gibco, #PHG0313
Recombinant Mouse EGF	-	-	-	50 ng/mL			Gibco, #PMG8041
FGF-10	100 ng/mL	100 ng/mL	100 ng/mL	-	100 ng/mL	-	Peprotech, #100-26-25
HGF	-	25 ng/mL	-	-	25 ng/mL	25 ng/mL	Peprotech, #100-39
Gastrin I human	1 nM	10 nM	-	10 nM	10 nM	10 nM	Sigma, #G9020
FGF-19	-	-	-	-	-	100 ng/mL	R&D, #969-FG-025
B27 Supplement (+ Vitamin A)	1X	-	1.50%	1X	-	1X	Invitrogen, #17504001
B27 Supplement (- Vitamin A)	-	1X	-	-	1X	-	Invitrogen, #12587001
A83-01	2 µM	5 µM	5 µM	0.5 µM	5 µM	0.5 µM	Tocris, #2939
PGE2	-	-	-	-	-	-	Tocris, #2296
N2 Supplement	-	1X	-	-	1X	1X	Gibco, #17502001
Forskolin	-	10 µM	-	-	10 µM	-	Tocris, #1099
Nicotinamide	10 mM	10 mM	-	10 mM	10 mM	-	Sigma, #N0636
SB202190	-	-	-	10 µM	-	-	Stem Cell Technologies, #72634
DAPT	-	-	-	-	-	10 µM	Sigma, #D5942

Medium component	Final concentration						Source
	Gastric	Liver	Kidney	Colon	Liver Starting	Liver Differentiation	
Dexamethasone	-	-	-	-	-	3 μM	Sigma, #D4902
BMP7	-	-	-	-	-	25 ng/mL	Peprotech, #OP1 120-03
hES Cell Cloning and Recovery	-	-	-	-	1X	-	Stemgent, #01-0014-500

Supplementary Table S3. DNA adduct levels in normal human tissue organoids after treatment with AFB_1, AAI, PhIP and *N*-OH-PhIP

Compound/ Adduct Type	Organoid Type		Concentration (µM)	Mean Adduct Level (adducts/10^7 nucleosides)	SD
AFB_1/AFB_1-FapyGua	Gastric	D95	6.25	38	10.31
			25	151	15.19
		D88	37.5	103	45.81
			150	342	39.89
	Liver	D4 Undifferentiated	37.5	1467	324.8
			150	5547	1133
		D4 Differentiated	37.5	1298	127.8
			150	2720	537.1
	Kidney	D50	37.5	383	155.0
			150	1080	198.2
		D21	37.5	426	77.84
			150	1177	47.13
AAI/dA-AL-I	Gastric	D95	12.5	56	13.68
			50	412	86.72
		D88	1.56	193	21.61
			6.25	843	81.15
	Kidney	D50	25	19	6.62
			200	1176	231.3
		D21	25	25	6.89
			200	1276	353.6
PhIP/dG-C8-PhIP	Gastric	D95	250	1.2	0.59
		D88	250	0.6	0.38
	Colon	D311	250	2.2	0.19
		D351	250	2.7	1.3
N-OH-PhIP/dG-C8-PhIP	Gastric	D95	2.5	21	1.34
			3.75	48	10.89
		D88	17.5	43	17.77
			20	57	28.95

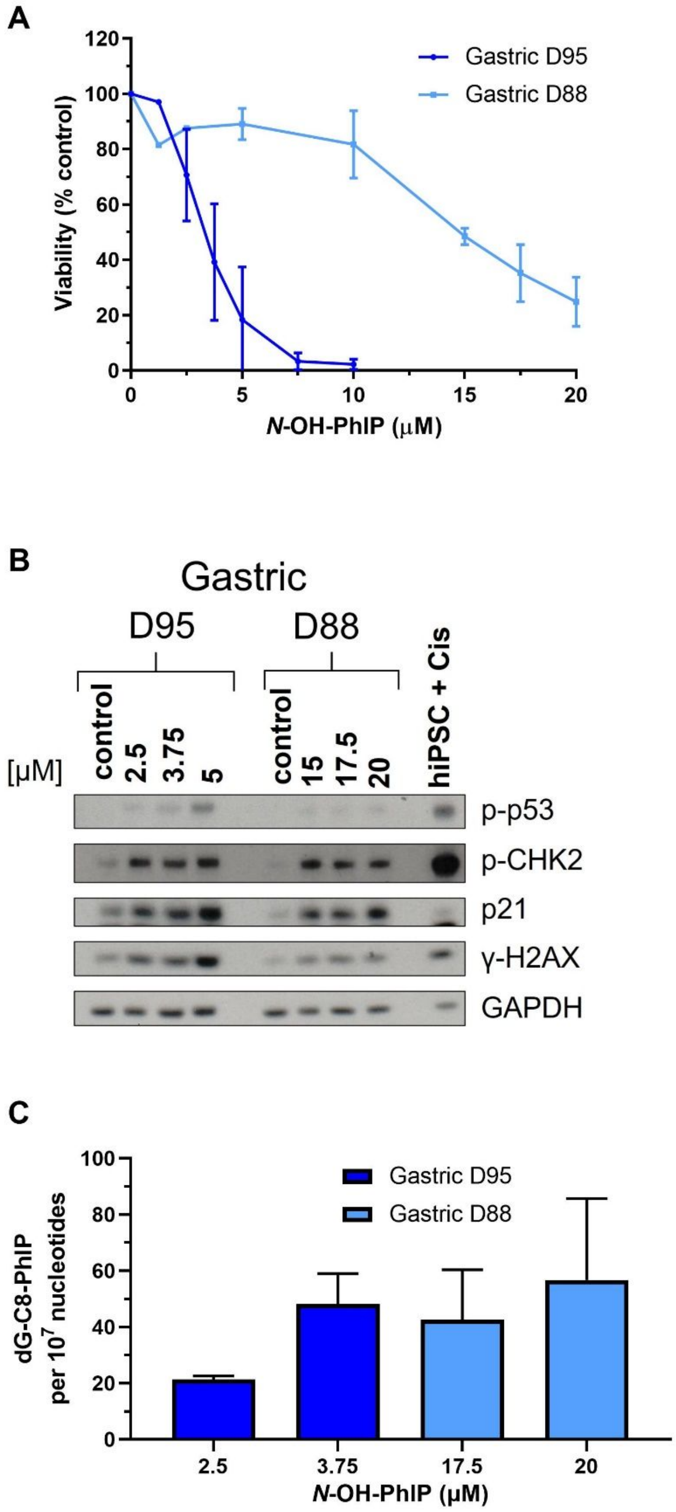

Supplementary Fig. S1. Organoids from normal human gastric tissues (D95 and D88) were treated with various concentrations of *N*-OH-PhIP for 48 hours. Vehicle control (DMSO) was included. (**A**) Cell viability (% control) was measured using the CellTiter-Glo assay. Results are shown as mean ± SD (n≥3). (**B**) Organoid lysates were analysed by Western blotting. Various DDR proteins (p-p53, p-CHK2, p21 and γ-H2AX) were detected and GAPDH was used as a loading control. iPSC + Cis (hiPSC treated with 3.125 μM cisplatin) was used as a positive control. Representative blots are shown (n=2). (**C**) dG-C8-PhIP DNA adduct formation was quantified using UPLC-ESI/MS^3. Results are shown as mean ± SD (n≥3).

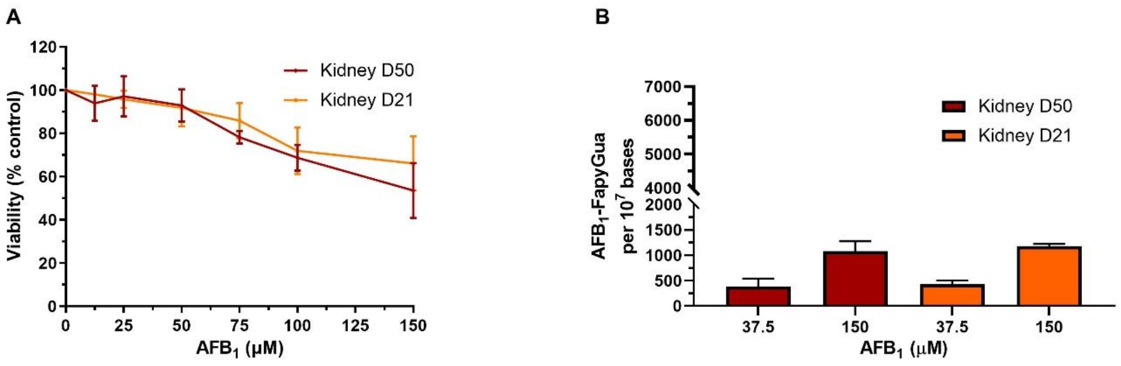

Supplementary Fig. S2. Organoids from normal human kidney tissues (D50 and D21) were treated with various concentrations of AFB_1 for 48 hours. Vehicle control (DMSO) was included. (**A**) Cell viability (% control) was measured using the CellTiter-Glo assay. Results are shown as mean ± SD (n≥3). (**B**) AFB_1-FapyGua adduct formation was quantified using LC-MS/MS. Results are shown as mean ± SD (n≥3).
